# *Simoponefisheri* sp. n., a new species of Dorylinae ants (Hymenoptera, Formicidae) from China, with an illustrated key to the *S.grandidieri*-group species

**DOI:** 10.3897/zookeys.838.29465

**Published:** 2019-04-11

**Authors:** Zhilin Chen, Yazhen Chen, Shanyi Zhou

**Affiliations:** 1 Key Laboratory of Ecology of Rare and Endangered Species and Environmental Protection (Guangxi Normal University), Ministry of Education, Guilin, 541004, China Guangxi Normal University Guilin China; 2 Guangxi Key Laboratory of Rare and Endangered Animal Ecology, Guangxi Normal University, Guilin, 541004, China Guangxi Normal University Guilin China; 3 College of Life Sciences, Guangxi Normal University, Guilin 541004, China Guangxi Normal University Guilin China

**Keywords:** *Simoponegrandidieri* group, new species, China

## Abstract

*Simoponefisheri***sp. n.**, a new species of the subfamily Dorylinae, is described based on the worker caste. The new species is separated easily from the other named congeners by the longitudinally striate sculpture on the posterolateral portion of pronotum. An illustrated key is presented to species of the *S.grandidieri* group based on the worker caste.

## Introduction

The genus *Simopone* was established by Forel (1891) based on the type species *Simoponegrandidieri* and assigned to Dorylinae by Emery (1895, 1901). Over the years it was considered as a member of the subfamily Ponerinae (Dalla Torre 1893; Forel 1893, 1917; Wheeler 1910, 1922; Emery 1911; Donisthorpe 1943; Brown 1975) or Cerapachyinae (Wheeler 1902; Bolton 1990, 1994, 2003). Brady et al. (2014) placed it into the subfamily Dorylinae again. The genus is an Old World lineage and 39 species have so far been described (Borowiec 2016; AntCat 2018). Brown (1975) first provided a key to Afrotropical species. Bolton and Fisher (2012) revised the genus globally, recognized 38 species, and proposed three species groups: *S.emeryi* group, *S.grandidieri* group, and *S.schoutedeni* group. Later, Chen et al. (2015) described one new species from Yunnan, China, which they assigned to the *S.grandidieri* group and included in a key to all known species of the *S.grandidieri* group. Other related taxonomic works were made by the following authors: Kutter (1976, 1977), Menozzi (1926), Taylor (1965, 1966), Emery (1899), Forel (1891, 1892), Santschi (1923), Arnold (1915, 1954), Radchenko (1993), and Weber (1949).

In the course of our recent survey of ants in Guangxi Daqingshan, southern China, we discovered a species that is clearly different from the known species of *Simopone*. We describe it as *S.fisheri* sp. n. and provide an updated key to the *S.grandidieri* group based on the worker caste.

## Materials and methods

The examination of the specimens was carried out by Leica M205A stereomicroscope. High-quality multifocal montage images were produced with Leica DFC 450 digital imaging system and Leica Application Suite v. 4.3 software. Standard measurements and indices follow Bolton and Fisher (2012). All measurements are expressed in millimeters.

**AIIL** Abdominal Segment II (petiole) Length: The maximum length of abdominal segment II (petiole), measured in dorsal view and including longitudinal projections of the posterolateral corners where such occur.

**AIIW** Abdominal Segment II (petiole) Width: The maximum width of abdominal segment II (petiole), measured in dorsal view but omitting laterally projecting teeth when such occur at the posterolateral corners.

**AIIIL** Abdominal Segment III Length: The maximum length of abdominal segment III (postpetiole), measured in dorsal view.

**AIIIW** Abdominal Segment III Width: The maximum width of abdominal segment III (postpetiole), measured in dorsal view.

**AIVL** Abdominal Segment IV Length: The maximum length of the posttergite of abdominal segment IV (first gastral), measured in dorsal view, omitting the pretergite.

**AIVW** Abdominal Segment IV Width: The maximum width of abdominal segment IV (first gastral), measured in dorsal view.

**CI** Cephalic Index: HW divided by HL, × 100.

**ED** Eye Diameter: The maximum diameter of the eye.

**EP** Eye Position Ratio: In full-face view, the distance from a horizontal line that intersects the mid-point of the anterior clypeal margin, or from a line that spans the anterior-most points of the frontal lobes (depending on which projects farthest forward), to the level of a line that spans the anterior margins of the eyes, divided by the horizontal distance from a line that spans the posterior margins of the eyes to one that spans the posterior corners of the head.

**HL** Head Length: The length of the head capsule excluding the mandibles; measured in full-face view in a straight line from the mid-point of the anterior clypeal margin or from a line that spans the anteriormost points of the frontal lobes (depending on which projects farthest forward) to the level of a line that spans the posterior corners of the head capsule. In species with a strongly reflexed true anterior clypeal margin (i.e. the clypeo-labral junction) the measurement is taken from the midpoint of the apparent margin as seen in full-face view.

**HW** Head Width: The maximum width of the head immediately behind the eyes, measured in full-face view.

**SI** Scape Index: SL divided by HW, × 100.

**TL** Total Length: The total outstretched length of the individual, from the mandibular apex to the gastral apex.

**SL** Scape Length: The maximum straight-line length of the scape, excluding the basal constriction or neck that occurs just distal of the condylar bulb.

**SW** Scape Width: The maximum width of the scape, usually at its apex. FCW-Frontal Carina Width: The distance across the maximum separation of the frontal lobes or frontal carinae (whichever is greatest), measured in full-face view.

**WL** Weber’s Length of Mesosoma (= Alitrunk Length): The diagonal length of the mesosoma in profile, from the angle at which the pronotal collar meets the neck to the posterior basal angle of the metapleuron.

The holotype worker and seven paratype workers are deposited in the Insect Collection of Guangxi Normal University (**GXNU**), Guilin, Guangxi, China, and one paratype worker will be deposited in the Insect Collection, Southwest Forestry University (**SWFU**), Kunming, Yunnan, China.

### A list of *Simoponegrandidieri*-group species

***S.bakeri* Menozzi, 1926**: 92. SINGAPORE.

[Non-type gyne images examined, CASENT0173045, photos by California Academy of Sciences, available on AntWeb.org].

***S.chapmani* Taylor, 1966**: 287. PHILIPPINES.

[Holotype worker images examined, CASENT0173044, photos by California Academy of Sciences, available on AntWeb.org].

***S.elegans* Bolton & Fisher, 2012**: 48. MADAGASCAR.

[Holotype worker images examined, AntWeb, CASENT0492213, photos by Shannon Hartman, available on AntWeb.org].

***Simoponefisheri* sp. n.** CHINA.

[Holotype worker and 8 paratype workers examined].

***S.grandidieri* Forel, 1891**: 141. MADAGASCAR.

[Holotype worker images examined, CASENT0101842, photos by April Nobile, available on AntWeb.org].

***S.gressitti* Taylor, 1965**: 3. NEW GUINEA.

[Holotype worker images examined, CASENT0249114, photos by Ryan Perry, available on AntWeb.org].

***S.laevissima* Arnold, 1954**: 291. UGANDA.

[No specimen and image examined].

***S.oculata* Radchenko, 1993**: 45. VIETNAM.

[Holotype worker images examined, CASENT0917355, photos by Kate Martynova, available on AntWeb.org].

***S.yunnanensis* Chen, Zhou & Liang, 2015**: 8. CHINA.

[Holotype worker examined].

Description

## Tahonomy

### Simopone fisheri sp. n.

Taxon classificationAnimaliaHymenopteraFormicidae

http://zoobank.org/0C2A62F4-CE26-4AA7-A135-3C27E763EDD4

#### Type material.

Holotype worker: CHINA, Guangxi, Longzhou County, bingqiao Town, Daqingshan, 22.297° N, 106.695° E, 500 m alt., evergreen broad-leaved forest, nest in a twig, hand collecting, 21.V.2016, Zhilin Chen leg., No. G160312. Paratypes: 8 workers from the same colony as the holotype.

#### Holotype worker.

**Figures 1–4.**
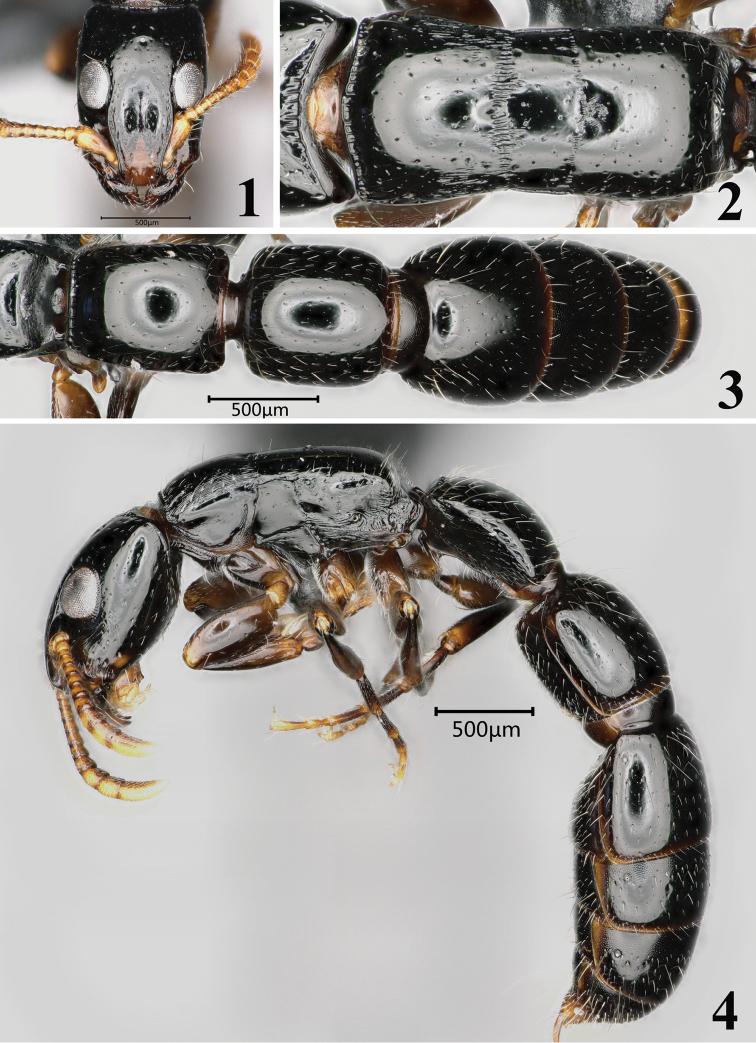
*Simoponefisheri* sp. n., holotype worker **1** head in full-face view **2** mesosoma in dorsal view **3** petiole and gaster in dorsal view **4** body in lateral view.

(Figs 1–4). AIIL 0.80, AIIW 0.68, AIIIL 0.75, AIIIW 0.74, AIVL 0.85, AIVW 0.86, CI 76, ED 0.29, EP 86, HL 1.06, HW 0.81, SI 46, SL 0.28, SW 0.13, TL 6.06, WL 1.45, AIIW/AIIL 0.85, AIIIW/AIIIL 0.99.

Head in full-face view nearly rectangular, longer than broad (CI 76–78), broadest around the level of eye; sides broadly weakly convex, but shallowly concave anterior to eyes; posterior margin concave; posterolateral corner forming a blunt angle. Mandibles subtriangular, with masticatory margin finely dentate. Clypeus without median carina; anterior margin of median portion of clypeus broadly rounded. Frontal carinae horizontal, widely separated by broad frontal area; outer margins of frontal lobe divergent posteriad and extending beyond to the anterior margins of eyes. Antennae 11-segmented; scape short, clavate, not reaching to anterior margin of eye. Antennal scrobe extending from antennal socket to the anterior margin of the eye. Eyes large, occupying about 1/3 length of the side of head; the center point of eye posterior to the mid-length of head; outer margin of eye in full-face view not touching the lateral margin of head. Median and lateral ocelli present, minute and closely approximated to each other.

Mesosoma in lateral view weakly convex on pronotum, with a weak concavity between pronotum and mesonotum. Pronotal disc in dorsal view with anterodorsal margin carinate and convex anteriad; humeri narrowly round (not sharply angulate); lateral margins weakly convergent posteriad. Promesonotal suture in dorsal view recognized as a narrow and longitudinally rugose band, slightly convex anteriad. Dorsolateral borders of pronotum and mesonotum not forming longitudinal carina. Metanotal groove in dorsal view as a very narrow band, slightly convex posteriad. Dorsum and posterior slope of propodeum in lateral view forming a round corner, without a carina between the two faces.

Petiole (AII) longer than broad (AIIW/AIIL = 0.85), with anterodorsal carina strong and straight, in dorsal view with sides divergent posteriorly, with posterolateral corner narrowly round; dorsum in lateral view continuously convex; posteroventral corner of subpetiolar process produced as an acute hook or spine. Postpetiole (AIII) as broad as long, a little longer than high, in lateral view with sides almost parallel; dorsum in lateral view moderately convex. A conspicuous girdling constriction present between AIV and AV.

Head scattered with minute piligerous punctures, with spaces between punctures smooth and shining; mesosoma largely smooth and shining, with sparse minute piligerous punctures, longitudinally striate on posterolateral portion of dorsal face of pronotum, central portion of lateral face of pronotum and most part of metapleuron smooth and shining; waist segments and gaster largely smooth and shining, with sparse minute piligerous punctures, finely reticulate on anterior portions of AV, AVI and AVII.

Body scattered with short and decumbent background hairs; sides of head with one or two long setae; inner margin of each eye posteriorly with two long setae posteriorly; scape with several suberect setae; antennal funiculi with abundant setae; anterior portion of mesosoma scattered with long suberect setae; petiole, postpetiole, tergite of AIV, posterior edges of AV and AVI, pygidium and hypopygium with abundant setae.

Body color black; antenna, trochanter, spur, apical portion of tarsi yellowish brown.

#### Paratype wokers.

AIIL 0.79–0.83, AIIW 0.66–0.69, AIIIL 0.72–0.77, AIIIW 0.72–0.75, AIVL 0.81–0.86, AIVW 0.85–0.87, CI 76–78, ED 0.29, EP 85–86, HL 1.04–1.06, HW 0.80–0.83, SI 44–46, SL 0.27–0.28, SW 0.12–0.13, TL 6.01–6.12, WL 1.45–1.49, AIIW/AIIL 0.82–0.85, AIIIW/AIIIL 0.97–0.99. Similar to holotype, with the following exceptions. The metanotal suture of one paratype specimen well developed but incomplete and another one paratype specimen faintly marked.

#### Etymology.

The new species is named in honor of Brian L. Fisher (California Academy of Sciences, United States of America) for his outstanding contributions to ant systematics.

#### Comparison notes.

This new species is the ninth species of the *S.grandidieri* species group and is morphologically most similar to *S.oculata*, but is easily differentiated from it by dorsolateral borders of pronotum round and not forming longitudinal carina. The new species is also similar to *S.yunnanensis* but is easily differentiated from it by dorsolateral portion of pronotum longitudinally striate and metanotal groove present.

The dorsolateral borders of pronotum in *S.yunnanensis* forms a right angle but never forms longitudinal carina; the original description of *S.yunnanensis* by Chen et al. (2015) needs to be corrected as above.

#### An illustrated key to species of the *Simoponegrandidieri*group based on the worker caste

The following key is built upon the key by Bolton﻿ and Fisher (2012).

**Table:** 

1	In full-face view, outer margins of eye just interrupting lateral margin of head (Figs 5–6)	**2**
–	In full-face view, outer margins of eye not interrupting lateral margin of head (at most toughing the lateral margin as seen in Fig. 7)	**4**
2	Frontal carina relatively short, ending far away from the level of the anterior margins of eyes; leading edge of scape without standing setae (Fig. 8)	*** S. grandidieri ***
–	Frontal carina relatively long, extending beyond the level of the anterior margins of eye; leading edge of scape with standing setae (Fig. 9)	**3**
3	Eyes located far back on head (EP 1.90)	*** S. laevi ssima ***
–	Eyes located slightly more anteriorly on head (EP 0.74–0.84)	*** S. elegans ***
4	Anterior margin of clypeus with a prominent tooth at its midpoint (Fig. 10)	*** S. bakeri ***
–	Anterior margin of clypeus broadly rounded, and without a tooth at its midpoint (Figs 11, 12).	**5**
5	AII almost as broad as long (AIIW/AIIL 0.96) (Fig. 13)	*** S. gressitti ***
–	AII distinctly longer than broad (AIIW/AIIL ≤ 0.86) (Fig. 14).	**7**
6	Head in full-face view distinctly trapezoidal; lateral tooth of clypeus inconspicuous (Fig. 15)	*** S. chapmani ***
–	Head nearly rectangular in full-face view (Figs 18, 19); lateral tooth of clypeus conspicuous (Figs 16–18)	**7**
7	Large species (TL ≥ 8.0 mm); maximum diameter of eye smaller than the minimum distance between eyes; posterolateral portion of dorsal face of pronotum striate longitudinally (Fig. 19)	***S.fisheri* sp. n.**
–	Medium-sized or small species (TL ≤ 6.5 mm); the maximum diameter of eye equal to the minimum distance between eyes; posterolateral portion of dorsal face of pronotum smooth and shining (Figs 20, 21).	**8**
8	Medium-sized species (TL = 6.5 mm); posterior margin of head distinct concave; lateral side of 1/3 posterior head gradually convergent (Fig. 22)	*** S. yunnanensis ***
–	Small species (TL = 5.5 mm); posterior margin of head almost straight; lateral side of 1/3 posterior head gradually divergent (Fig. 23).	*** S. oculata ***

**Figures 5–7.**
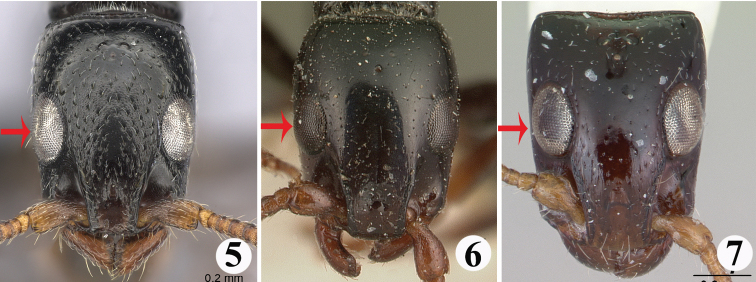
Head in full-face view of the worker of *Simopone* spp. **5***S.elegans*, type (specimen CASENT0492213; photo by Shannon Hartman, available on AntWeb.org) **6***S.grandidieri*, type (specimen CASENT0101842; photo by April Nobile, available on AntWeb.org) **7***S.chapmani*, type (specimen CASENT0173044; photo by April Nobile, available on AntWeb.org).

**Figures 8–9.**
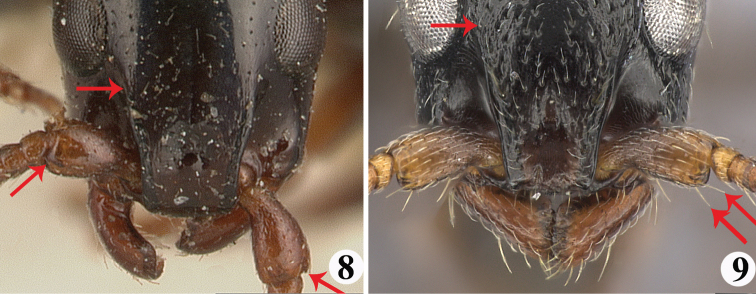
Head in full-face view of the worker of *Simopone* spp. **8***S.grandidieri*, type (specimen CASENT0101842; photo by April Nobile, available on AntWeb.org) **9***S.elegans* type (specimen CASENT0492213; photo by Shannon Hartman, available on AntWeb.org).

**Figures 10–12.**
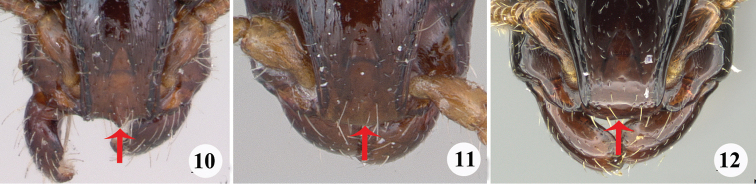
Head in full-face view **10***S.bakeri* gyne (specimen CASENT0173045; photo by April Nobile, available on AntWeb.org) **11***S.chapmani* type (specimen CASENT0173044; photo by April Nobile, available on AntWeb.org) **12***S.gressitti* type (specimen CASENT0249114; photo by Ryan Perry, available on AntWeb.org).

**Figures 13, 14.**
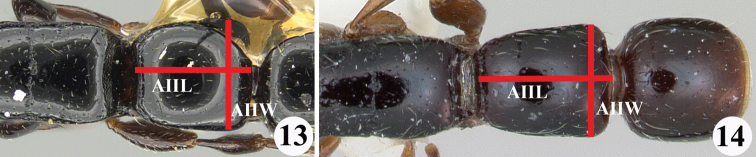
Petiole (AII) in dorsal view of the worker of *Simopone* spp. **13***S.gressitti*, type (specimen CASENT0249114; photo by Ryan Perry, available on AntWeb.org) **14***S.chapmani*, type (specimen CASENT0173044; photo by April Nobile, available on AntWeb.org).

**Figures 15–18.**
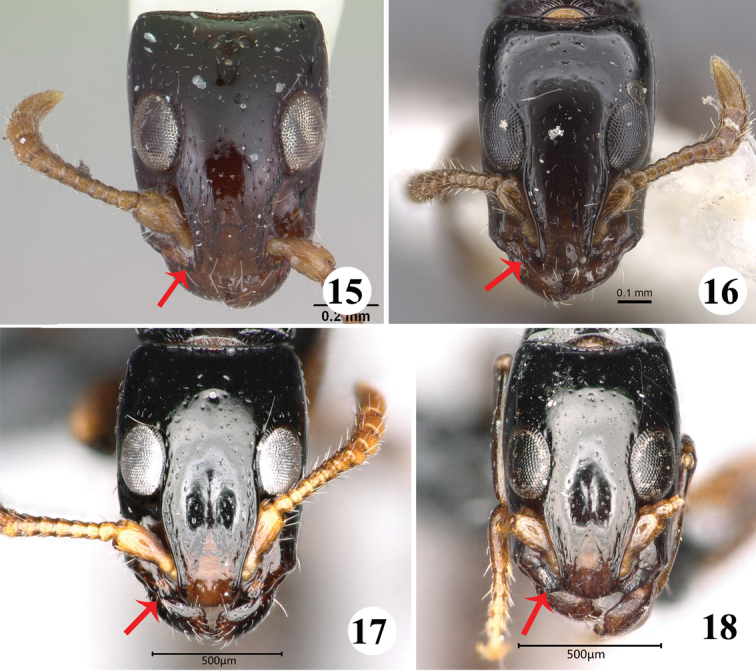
Head in full-face view of the worker of *Simopone* spp. **15***S.chapmani*, type (specimen CASENT0173044; photo by April Nobile, available on AntWeb.org) **16***S.oculata*, type (specimen CASENT0917355; photo by Kate Martynova, available on AntWeb.org) **17***S.fisheri*, type (photo by Zhlin Chen) **18***S.yunnanensis* type (photo by Zhlin Chen).

**Figures 19–21.**
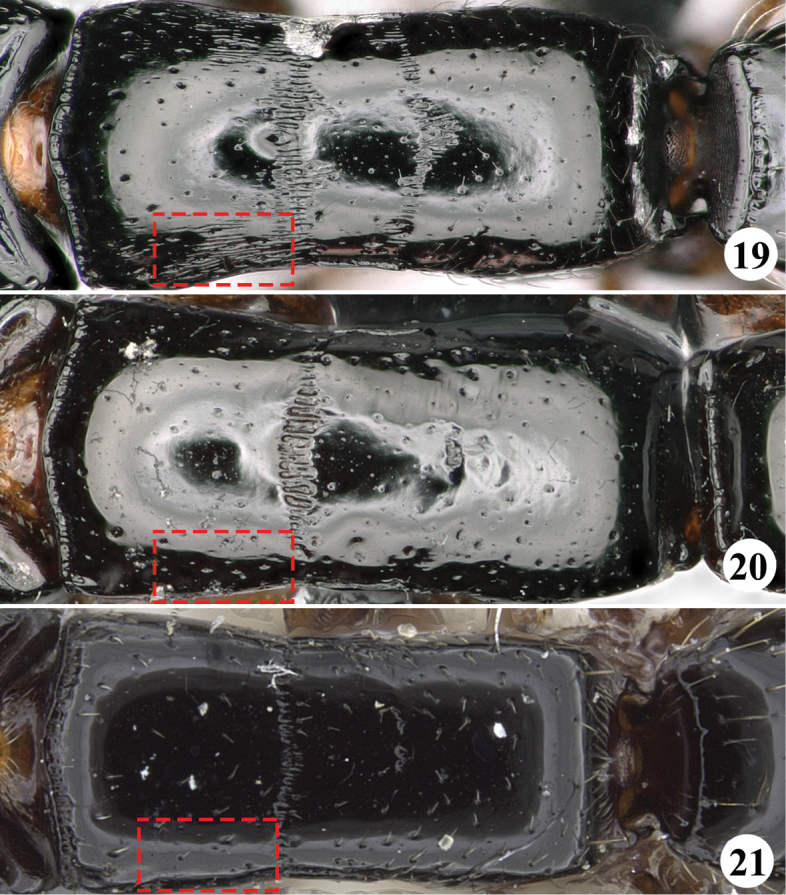
Mesosoma in dorsal view of the worker of *Simopone* spp. **19***S.fisheri*, type (photo by Zhlin Chen) **20***S.yunnanensis*, type (photo by Zhlin Chen) **21***S.oculata*, type (specimen CASENT0917355; photo by Kate Martynova, available on AntWeb.org).

**Figures 22–23.**
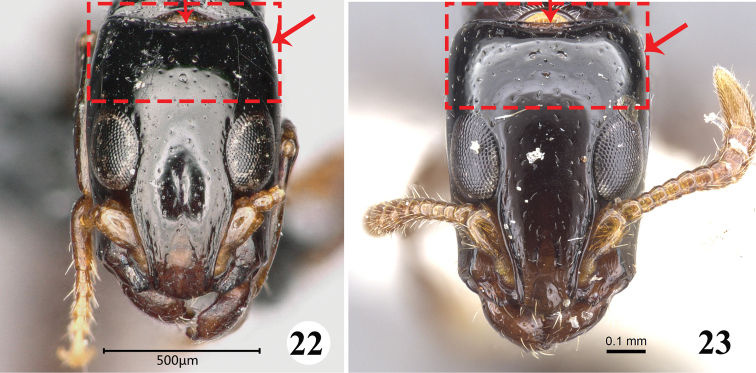
Head in full-face view of the worker of *Simopone* spp. **22***S.yunnanensis*, type (photo by Zhlin Chen) **23***S.oculata*, type (specimen CASENT0917355; photo by Kate Martynova, available on AntWeb.org).

## Supplementary Material

XML Treatment for Simopone fisheri
